# Influence of Breast Density and Menopausal Status on Background Parenchymal Enhancement in Contrast-Enhanced Mammography: Insights from a Retrospective Analysis

**DOI:** 10.3390/cancers17010011

**Published:** 2024-12-24

**Authors:** Luca Nicosia, Luciano Mariano, Carmen Mallardi, Adriana Sorce, Samuele Frassoni, Vincenzo Bagnardi, Cristian Gialain, Filippo Pesapane, Claudia Sangalli, Enrico Cassano

**Affiliations:** 1 Division of Breast Radiology, Department of Medical Imaging and Radiation Sciences, European Institute of Oncology, IRCCS, 20141 Milan, Italy; filippo.pesapane@ieo.it (F.P.); enrico.cassano@ieo.it (E.C.); 2 Postgraduation School in Radiodiagnostics, Università degli Studi di Milano, Via Festa del Perdono, 7, 20122 Milan, Italy; carmen.mallardi@unimi.it (C.M.); adriana.sorce@unimi.it (A.S.); 3 Department of Statistics and Quantitative Methods, University of Milano-Bicocca, 20126 Milan, Italy; samuele.frassoni@unimib.it (S.F.); vincenzo.bagnardi@unimib.it (V.B.); 4 Department of Medicine and Surgery, University of Milano-Bicocca, 20126 Milan, Italy; 5 Clinical Trial Office, European Institute of Oncology IRCCS, 20141 Milan, Italy; cristian.gialain@ieo.it (C.G.); claudia.sangalli@ieo.it (C.S.)

**Keywords:** background parenchymal enhancement, contrast-enhanced mammography, breast density, menopausal status

## Abstract

**Background:** Contrast-enhanced mammography (CEM) has recently gained recognition as an effective alternative to breast magnetic resonance imaging (MRI) for assessing breast lesions, offering both morphological and functional imaging capabilities. However, the phenomenon of background parenchymal enhancement (BPE) remains a critical consideration, as it can affect the interpretation of images by obscuring or mimicking lesions. While the impact of BPE has been well-documented in MRI, limited data are available regarding the factors influencing BPE in CEM and its relationship with breast cancer (BC) characteristics. **Materials:** This retrospective study included 116 patients with confirmed invasive BC who underwent CEM prior to biopsy and surgery. Data collected included patient age, breast density, receptor status, tumor grading, and the Ki-67 proliferation index. BPE was evaluated by two radiologists using the 2022 ACR BI-RADS lexicon for CEM. Statistical analyses were conducted to assess the relationship between BPE, patient demographics, and tumor characteristics. **Results:** The study found a significant association between higher levels of BPE and specific patient characteristics. In particular, increased BPE was more commonly observed in patients with higher breast density (*p* < 0.001) and those who were pre-menopausal (*p* = 0.029). Among patients categorized under density level B, the majority exhibited minimal BPE, while those in categories C and D showed progressively higher levels of BPE, indicating a clear trend correlating higher breast density with increased enhancement. Additionally, pre-menopausal patients demonstrated a higher likelihood of moderate to marked BPE compared to post-menopausal patients. Despite these significant associations, the analysis did not reveal a meaningful correlation between BPE intensity and tumor subtypes (*p* = 0.77) or tumor grade (*p* = 0.73). The inter-reader agreement for BPE assessment was substantial, as indicated by a weighted kappa of 0.78 (95% CI: 0.68–0.89), demonstrating consistent evaluation between radiologists. **Conclusions:** These findings suggest that BPE in CEM is influenced by factors like breast density and age, aligning with patterns observed in MRI studies. However, BPE intensity was not associated with tumor subtypes or grades, indicating a poorer prognosis. These insights highlight the potential of BPE as a risk biomarker in preventive follow-up, particularly for patients with high breast density and pre-menopausal status. Further multicentric and prospective studies are needed to validate these results and deepen the understanding of BPE’s role in CEM diagnostics.

## Simple Summary

This study investigates background parenchymal enhancement (BPE) in contrast-enhanced mammography (CEM) and its association with breast density, menopausal status, and breast cancer (BC) characteristics. Conducted retrospectively on 116 patients with invasive BC, the research reveals that BPE is higher in pre-menopausal women and those with greater breast density. These findings align with observations in MRI studies. However, BPE intensity showed no correlation with tumor subtype or grade, suggesting it does not reflect poorer prognostic indicators. This study highlights the potential of BPE as a risk biomarker, particularly for preventive follow-up in high-density or pre-menopausal patients. Further multicentric and prospective studies are recommended to validate these findings and explore the role of BPE in CEM diagnostics.

## 1. Introduction

To provide an increasingly precise and personalized diagnostic approach in breast imaging, contrast-enhanced mammography (CEM) has recently emerged as a viable alternative to breast magnetic resonance imaging (MRI) for various clinical indications [1,2] due to its ability to provide both morphological and functional assessment based on the use of an iodinated contrast agent [3,4].

In this context, it is necessary to consider and address the background parenchymal enhancement (BPE) phenomenon in routine breast imaging analysis. BPE refers to the physiological uptake of contrast agents by the breast parenchyma during contrast-enhanced imaging [5,6]. Elevated BPE can complicate image interpretation by potentially obscuring underlying lesions, which may lead to false negative findings, or mimicking malignancy, resulting in false positives [7,8,9]. It is hypothesized that a high degree of BPE may pose greater challenges in CEM interpretation than MRI, primarily due to CEM’s two-dimensional imaging, which can result in overlapping fibroglandular tissue [10,11,12].

The relationship between BPE and specific hormonal and patient constitutional characteristics remains largely unknown and has predominantly been investigated in the context of breast MRI. For instance, BPE has been linked to fibroglandular tissue volume and the aging process [13]. Additionally, BPE may be associated with hemoglobin levels, hematocrit, and applied mammographic pressure [6]. BPE is also affected by hormonal changes over a woman’s lifetime, such as those associated with the menstrual cycle, endocrine therapy, etc. [14,15,16].

One of the most significant biological markers that affects background parenchymal enhancement (BPE) in contrast-enhanced imaging is hormonal changes:

- Estrogen and progesterone influence: BPE levels in the breast tend to be higher in pre-menopausal women and are regulated by the cyclical levels of estrogen and progesterone [14]. These hormones potentiate vascular permeability and the activity of glands and cause greater enhancement on imaging. BPE is generally less in post-menopausal women, owing to decreased hormonal activity [14,15,16].
- Exogenous hormones and clinical implications: The increase in BPE associated with HRT in post-menopausal women and contraceptive use by younger women shows the importance of exogenous hormones for BPE. This has also been observed in studies showing increased BPE amongst women who were undergoing HRT as compared to women not on HR [17,18].

These observations underline the importance of considering hormonal status and therapies when interpreting BPE in breast imaging. Adjustments in imaging timing (e.g., during specific phases of the menstrual cycle) can also help standardize BPE readings for better diagnostic accuracy [9].

Moreover, recent studies have confirmed that BPE is an independent predictor of BC risk, with higher BPE levels correlating with increased risk [19,20,21,22,23].

While the factors influencing BPE in MRI are well documented, those affecting BPE in CEM remain primarily unexplored [5]. Only a few studies have examined the association between CEM BPE and variables like age, breast density, or menopausal status [8,10,24]. Notably, a structured framework for describing and reporting BPE in CEM was recently introduced with the 2022 supplement to the American College of Radiology Breast Imaging Reporting and Data System (ACR BI-RADS) Atlas from 2013 [25]. Nonetheless, a comprehensive understanding of the relationship between CEM BPE and BC risk or menopausal status, as well as its association with histological features of BC, remains incomplete.

This study explores the potential correlation between BPE CEM and BC receptor profiles, representing what we believe to be the first investigation of this relationship. Additionally, we aim to examine the possible association between BPE, age, and breast density. The findings could provide valuable insights that enhance preoperative staging and inform the overall management of BC patients.

## 2. Materials and Methods

This retrospective monocentric study was approved by the Ethics Committees of the European Institute of Oncology (approval number UID 4264; approval date: 8 May 2024). Due to its retrospective design, the committee waived individual patient consent requirements.

The study cohort was drawn from patients presenting with a suspicious breast lesion who underwent CEM before undergoing a biopsy and subsequent surgery. Only patients with a confirmed histological diagnosis of invasive BC were included in the analysis.

Inclusion criteria:

- Patients with suspicious breast lesions (BIRADS ≥ 4) who had undergone CEM before histological assessment;
- Patients with a histological diagnosis of invasive BC;
- Patients who underwent breast surgery at the same institute.

Exclusion criteria:

- Patients whose histological results did not confirm invasive neoplasia;
- Patients who did not undergo surgery at our institute;
- Patients with images of inadequate quality

For the selected patients, data were collected on age at the time of the examination, lesion side and site, BI-RADS classification, breast density (assessed per ACR criteria), receptor status, and lesion grade. Surgical outcomes were also recorded, including histological grade, receptor status, and the Ki-67 proliferation index. Immunohistochemical analyses conducted in the pathology department provided data on the expression of Estrogen Receptor (ER), Progesterone Receptor (PR), Human Epidermal Growth Factor Receptor 2 (HER2), and Ki-67 antigen. Tumor grading followed the standard classification: G1 (low grade), G2 (intermediate grade), and G3 (high grade). Patients were further stratified based on their receptor profile [26].

Using recombined CEM images, BPE was independently evaluated by two breast radiologists (L.M., with 3 years of experience, and A.B., with 35 years of experience). BPE was classified as minimal, mild, moderate, or marked, following the 2022 ACR BI-RADS lexicon for CEM interpretation [25]. For analysis, the assessment of the more experienced radiologist was used.

All CEM examinations were performed with a full-field Digital Mammography system (Pristina™ Mammographer, GE Healthcare, Chalfont St. Giles, UK), modified for dual-energy imaging and equipped with specialized image acquisition and processing software. Before breast compression, patients received an automated intravenous injection of the iodinated contrast agent (Iohexol, 300 mg/mL, 1.5 mL/kg, Omnipaque^®^, GE Healthcare) at a flow rate of 3 mL/s. Two minutes after the end of the contrast injection, bilateral craniocaudal (CC) and mediolateral oblique (MLO) views were acquired, starting from the suspicious. Imaging was completed within five minutes. Each breast was imaged using two exposures: one at low energy (26–32 kVp) and one at high energy (45–49 kVp), which were subsequently combined to enhance the visualization of contrast uptake.

### Statistical Analysis

Continuous data are reported as median and ranges. Categorical data are reported as counts and percentages. Agreement among two radiologists in the classification of CEM background parenchymal enhancement was assessed using the weighted kappa statistic, with 95% CI. The chi-square test (or Fisher’s exact test, when the expected frequencies in any cell of the contingency table are less than 5) was used to evaluate the association between the more experienced radiologist’s background evaluation with subtype, grading, density, and age.

All reported *p*-values are two-sided, with a *p*-value less than 0.05 considered statistically significant. All analyses were performed with the statistical software SAS 9.4 (SAS Institute, Cary, NC, USA).

## 3. Results

**Figure 1:**
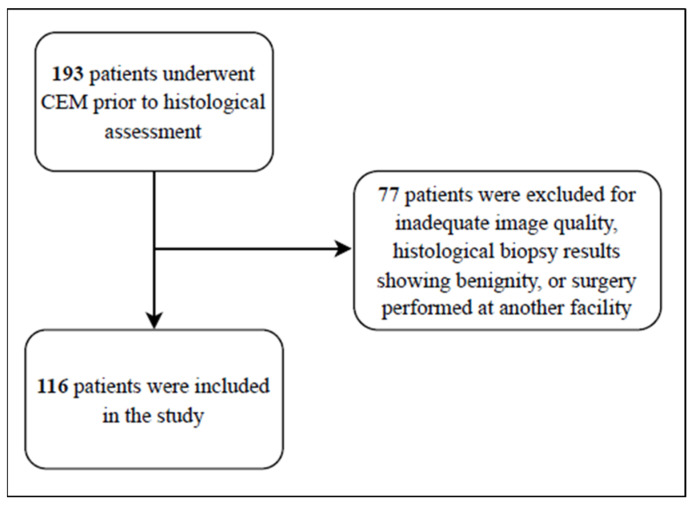
Flowchart of the study.

Out of an initial group of 193 patients who underwent CEM before histological assessment, 77 were excluded, leaving a final cohort of 116 patients enrolled in the study (Figure 1, CONSORT diagram).

The median age at the time of examination was 49 years (range: 28–83). Most lesions (94%) appeared as masses, and a BI-RADS 4c suspicion grade was assigned in 40.5% of cases. Tumors were graded as G2 in 44.7% of cases, while 58.6% exhibited high expression levels of estrogen and progesterone receptors. Additionally, 63.8% of cases had a Ki-67 proliferation index greater than 20%. Most patients (83.6%) were HER2-negative, and the most common tumor subtype was luminal B (Ki-67 ≥ 20%), accounting for 37.1% of cases.

A summary of the patients’ clinical characteristics is provided in Table 1.

For classifying CEM BPE into four categories, the inter-reader agreement between the two radiologists was 86.2%, with concordant evaluations in 100 out of 116 cases (Table 2). A substantial level of agreement was noted, as indicated by a weighted kappa statistic of 0.78.

The BPE values assigned by the more experienced radiologist were used for subsequent analyses. No significant correlation was found between tumor subtype and BPE (*p*-value = 0.77) or between BPE and tumor grading (*p*-value = 0.73). Details of these analyses are presented in Table 3 and Table 4.

**Figure 2:**
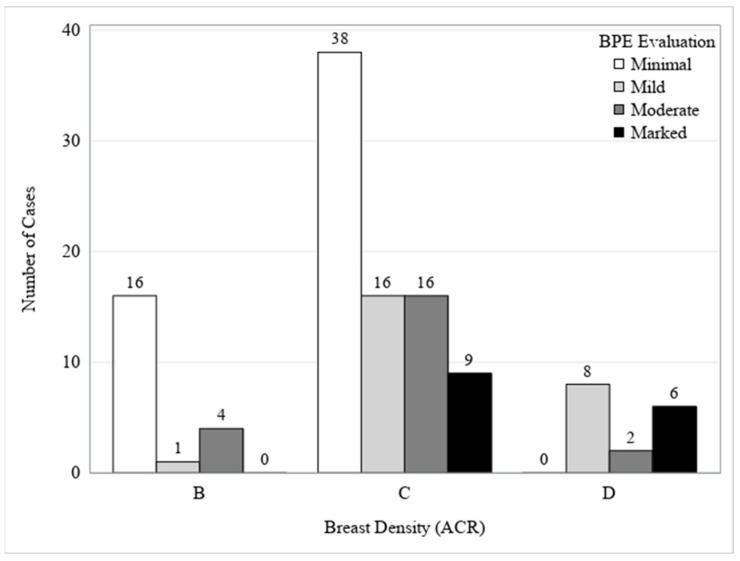
Association between physician 2 BPE evaluation and density (ACR) (*N* = 116).

There was a statistically significant correlation between higher breast density and higher BPE (*p*-value < 0.001), as well as between pre-menopausal age and higher BPE (*p*-value = 0.029). A different distribution of BPE across the three breast density levels was related. Among patients with density category B, 76% exhibited minimal BPE, and none had marked BPE. In category C patients, just under half had minimal BPE, 20% had mild, another 20% had moderate, and 11% had marked BPE. For patients in density category D, none showed minimal BPE; 50% had mild, 13% had moderate, and 38% exhibited marked BPE. Therefore, as breast density progresses from B to D, there is an apparent increase in the likelihood of higher BPE (Table 5 and Figure 2). 

A different distribution of BPE was also noted between the two age groups. Among patients under 50 years, 34% showed minimal BPE, while in the over-50 age group, the proportion of patients with minimal BPE was significantly higher at 61%. This suggests that older age is associated with a greater likelihood of minimal BPE (Table 6).

## 4. Discussion

BPE is a topic of significant debate in the context of breast MRI [7,27]. Several studies have demonstrated its substantial impact on image interpretation, as BPE can increase false-positive results by simulating malignancies and false-negative outcomes due to lesion masking [7]. Many factors influencing BPE in MRI have been studied, notably hormonal status, menstrual cycle, and fibroglandular tissue volume (breast density) [15,16].

The positive correlation between BPE, breast density and pre-menopausal status is an anticipated finding, given the known association between breast density and menopausal state [15,16]. The natural decrease in breast density with age, particularly during the menopausal transition, coincides with the reduction in circulating reproductive hormones [16]. In this context, CEM is gaining rapid acceptance in clinical practice due to its strong diagnostic performance comparable to MRI, faster procedure time, lower cost, and high patient tolerability [28].

Thus, an adequate knowledge of the phenomenon of BPE within the context of CEM would be crucial to optimally appraise its consequences and limit the risk of unwanted impairment on diagnostic correctness. This knowledge may assist with recognizing the patient subgroups that would benefit the most from this imaging method.

As the pathophysiological mechanisms of contrast enhancement are believed to be similar for both CEM and MRI, it is conceivable that many of the factors that play a role in BPE in MRI would also play a role in CEM BPE [29]. However, data on BPE in CEM are limited in the literature [8,10,24]. Our study, along with other limited studies, supports this hypothesis and illustrates a strong association of higher breast density, pre-menopausal age, and increasing BPE, similar to MRI findings [10]. This knowledge contributes to the identification of patients in whom BPE is less likely to affect diagnostic imaging, ensuring the accuracy of the tests made.

Moreover, identifying simple biomarkers such as BPE is becoming increasingly important in diagnostic practice, as it can help identify patients at higher risk for BC [30,31,32,33,34,35]. This strategy enhances targeted surveillance, potentially improving early detection and treatment outcomes. Elevated BPE may indicate breast areas are more susceptible to estrogen-driven malignant transformation, as observed in several MRI studies [30,31]. Emerging evidence suggests that higher CEM BPE levels could be linked to an increased risk of developing BC during long-term preventive follow-up [31,32,33,34,35].

To our knowledge, no prior research has specifically examined the relationship between BPE and receptor status in breast tumors, as explored in our study. Our analysis found no significant association between CEM BPE and histological cancer type or tumor grade. Additionally, patients with high BPE did not show an increased risk of BC with poor prognostic indicators. These findings are consistent with studies that have evaluated BPE and the relationship with receptor status in MRI [36,37].

Based on our results and aligning with the limited existing data, we hypothesize that BPE in CEM and MRI exhibits similar characteristics and is influenced by factors such as breast density and age, especially when distinguishing between pre- and post-menopausal women. This is crucial for optimizing patient selection to improve diagnostic performance. Our data, in line with MRI studies, do not show an association of the extent of BPE with receptor status linked to poor prognosis in BC patients. In contrast, our data, corroborated by MRI studies, demonstrate that BPE was not associated with receptor status linked to poorer prognosis in BC patients.

Using BPE patterns, clinicians can optimize risk-based imaging protocols, allowing tailored surveillance and interventional approaches. In addition to cancer risk, BPE in CEM may be a marker of systemic estrogen activity, providing a more comprehensive understanding of a patient’s hormonal milieu [22].

Regarding cancer risk, however, BPE in CEM potentially represented a systemic signature of estrogen activity, providing insight into a patient’s systemic hormones [22]. The knowledge can facilitate multidisciplinary care, especially among patients at high risk, as it integrates the role of imaging with hormonal and clinical assessment for tailored prevention and treatment strategies [38,39].

**Figure 3:**
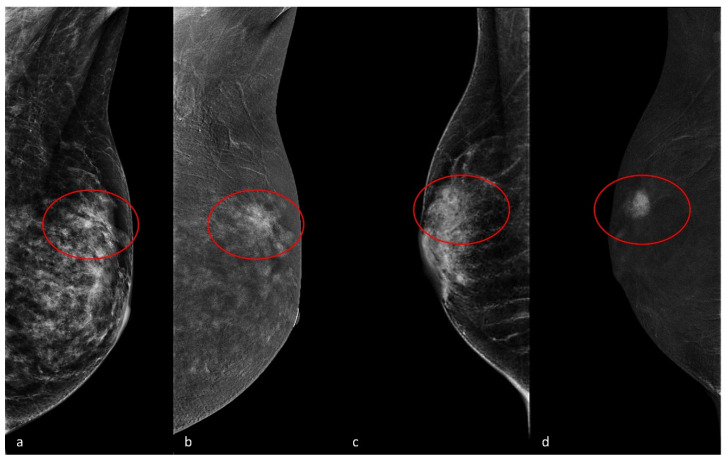
(**a**,**b**): Mediolateral oblique view shows a pseudo-distortive area (2D image, (**a**)) with corresponding enhancement (recombined image, (**b**)) in the upper sectors of the left breast. High mammary density (ACR BI-RADS c) and marked background enhancement can be appreciated. At biopsy, the lesion was a Grade 1 in situ ductal carcinoma, Luminal A. (**c**,**d**): Lateral view shows an irregular opacity (2D image, (**c**)) with corresponding enhancement (recombined image, (**d**)) in the upper periareolar sectors of the right breast. Low mammary density (ACR BI-RADS b) and minimal background enhancement can be appreciated. At biopsy, the lesion was a Grade 3 invasive ductal carcinoma, triple-negative.

Some typical examples of different levels of breast density and background are shown in Figure 3.

This study has limitations, including its retrospective and single-center design. Additionally, it did not account for menstrual cycle phases during imaging, which could influence BPE. Given the retrospective nature of the study, it was also not possible to retrieve relevant data regarding hormonal or other medical therapies, hematocrit levels, and the pressure applied during mammography. These data could be interesting and useful, based on the existing literature, for designing a prospective study.

## 5. Conclusions

Our study highlighted a correlation between CEM BPE intensity and factors such as breast density and pre-menopausal status, corroborating similar observations reported in MRI studies. However, no significant association was found between BPE and tumor receptor profiles or lesion pathological grading, suggesting that BPE intensity is not indicative of tumor characteristics with poorer prognosis. Further research, preferably prospective and multicentric, is needed to confirm these observations and better understand the implications of BPE in CEM diagnostic practice.

## Figures and Tables

**Table 1:** Patients’ demographic and tumor characteristics (*N* = 116).

Variable	Level	Overall (*N* = 116)
Age at radiological exam, median (min-max)		49 (28–83)
Type of lesion, *N* (%)	Mass	109 (94.0)
	Microcalcifications	5 (4.3)
	Mass with microcalcifications	2 (1.7)
Side, *N* (%)	Left	58 (50.0)
	Right	58 (50.0)
Quadrant, *N* (%)	Lower	24 (20.7)
	Middle	25 (21.6)
	Upper	67 (57.8)
BIRADS, *N* (%)	4b	25 (21.6)
	4c	47 (40.5)
	5	44 (37.9)
Grading, *N* (%)	G1	16 (14.0)
	G2	51 (44.7)
	G3	47 (41.2)
	Missing	2
ER\PGR, *N* (%)	Not expressed (Both 0)	17 (14.7)
	Incompletely expressed (ER < 50 or PgR < 50)	31 (26.7)
	Highly expressed (ER ≥ 50 and PgR ≥ 50)	68 (58.6)
Ki-67, median (min-max)		23 (3–90)
Ki-67, *N* (%)	<20%	42 (36.2)
	≥20%	74 (63.8)
HER2, *N* (%)	Not expressed	97 (83.6)
	Positive	19 (16.4)
Subtype, *N* (%)	Luminal A	42 (36.2)
	Luminal B (Ki-67 ≥ 20%)	43 (37.1)
	Luminal B (HER2 positive)	14 (12.1)
	HER2 positive	5 (4.3)
	Triple-negative	12 (10.3)
Density (ACR), *N* (%)	B	21 (18.1)
	C	79 (68.1)
	D	16 (13.8)

**Table 2:** Agreement between BPE evaluation of physicians 1 and 2 (*N* = 116).

Background		Physician 2				
		Minimal	Mild	Moderate	Marked	Total (%)
Physician 1	Minimal	54	0	3	2	59 (51)
	Mild	0	21	2	2	25 (22)
	Moderate	0	1	17	3	21 (18)
	Marked	0	3	0	8	11 (9)
	Total (%)	54 (47)	25 (22)	22 (19)	15 (13)	116 (100)

**Table 3:** Association between physician 2 BPE evaluation and subtype (*N* = 116).

	Subtype					
Background (Physician 2)	Luminal A	Luminal B (Ki-67 ≥ 20%)	Luminal B (HER2 +)	HER2 +	Triple-Negative	*p*-Value
	*N* (% col)					
Minimal	22 (52)	21 (49)	4 (29)	3 (60)	4 (33)	0.77
Mild	8 (19)	9 (21)	3 (21)	1 (20)	4 (33)	
Moderate	6 (14)	7 (16)	6 (43)	1 (20)	2 (17)	
Marked	6 (14)	6 (14)	1 (7)	0 (0)	2 (17)	

**Table 4:** Association between physician 2 BPE evaluation and grading (*N* = 114).

		Grading		
Background (Physician 2)	G1	G2	G3	*p*-Value
	*N* (% col)			
Minimal	10 (63)	21 (41)	21 (45)	0.73
Mild	1 (6)	12 (24)	12 (26)	
Moderate	3 (19)	11 (22)	8 (17)	
Marked	2 (13)	7 (14)	6 (13)	

**Table 5:** Association between physician 2 BPE evaluation and density (ACR) (N = 116).

		Density (ACR)		
Background (Physician 2)	B	C	D	*p*-Value
	*N* (% col)			
Minimal	16 (76)	38 (48)	0 (0)	<0.001
Mild	1 (5)	16 (20)	8 (50)	
Moderate	4 (19)	16 (20)	2 (13)	
Marked	0 (0)	9 (11)	6 (38)	

**Table 6:** Association between physician 2 BPE evaluation and age (*N* = 116).

	Age		
Background (Physician 2)	<50	>50	*p*-Value
	*N* (% col)		
Minimal	21 (34)	33 (61)	0.029
Mild	16 (26)	9 (17)	
Moderate	14 (23)	8 (15)	
Marked	11 (18)	4 (7)	

## Data Availability

The data presented in this study are available on request from the corresponding author. The data are not publicly available due to privacy concerns, in accordance with GDPR.
